# Bacterial Microbiota Profiling in Gastritis without *Helicobacter pylori* Infection or Non-Steroidal Anti-Inflammatory Drug Use

**DOI:** 10.1371/journal.pone.0007985

**Published:** 2009-11-24

**Authors:** Xiao-Xing Li, Grace Lai-Hung Wong, Ka-Fai To, Vincent Wai-Sun Wong, Larry Hin Lai, Dorothy Kai-Lai Chow, James Yun-Wong Lau, Joseph Jao-Yiu Sung, Chunming Ding

**Affiliations:** 1 Stanley Ho Centre for Emerging Infectious Diseases, The Chinese University of Hong Kong, Hong Kong, China; 2 Institute of Digestive Disease, The Chinese University of Hong Kong, Hong Kong, China; 3 Department of Anatomical and Cellular Pathology, The Chinese University of Hong Kong, Hong Kong, China; 4 Singapore Institute for Clinical Sciences, Agency for Science, Technology and Research, Singapore, Singapore; University of Hyderabad, India

## Abstract

Recent 16S ribosomal RNA gene (rRNA) molecular profiling of the stomach mucosa revealed a surprising complexity of microbiota. *Helicobacter pylori* infection and non-steroidal anti-inflammatory drug (NSAID) use are two main contributors to gastritis and peptic ulcer. However, little is known about the association between other members of the stomach microbiota and gastric diseases. In this study, cloning and sequencing of the 16S rRNA was used to profile the stomach microbiota from normal and gastritis patients. One hundred and thirty three phylotypes from eight bacterial phyla were identified. The stomach microbiota was found to be closely adhered to the mucosa. Eleven *Streptococcus* phylotypes were successfully cultivated from the biopsies. One to two genera represented a majority of clones within any of the identified phyla. We further developed two real-time quantitative PCR assays to quantify the relative abundance of the Firmicutes phylum and the *Streptococcus* genus. Significantly higher abundance of the Firmicutes phylum and the *Streptococcus* genus within the Firmicutes phylum was observed in patients with antral gastritis, compared with normal controls. This study suggests that the genus taxon level can largely represent much higher taxa such as the phylum. The clinical relevance and the mechanism underlying the altered microbiota composition in gastritis require further functional studies.

## Introduction

Commensal microbiota is an integral part of a human being [1]. The vast majority of microbes inhabit our gastrointestinal tract, with more than 800 species from nine bacterial and one archaeal phyla. This diverse microbiota contributes to gut maturation [2], [3], [4], host nutrition and pathogen resistance [5]. Microbes also directly interact with human host by regulating intestinal epithelial proliferation, fat storage and inflammatory responses [3], [6], [7]. While some microbes can single-handedly cause serious illness, many chronic conditions are probably due to perturbations of the overall microbiota. For example, allergies and asthma are linked to childhood antibiotic use which may alter intestinal microbiota [8]. Other conditions associated with intestinal microbiota include late-onset autism [9], inflammatory bowel disease [10], and cancer [11].

Traditionally, cultivation-based methods are used to obtain microbial isolates for further characterization. Such studies provided the foundation of our understanding of microbiology. However, cultivation is often labor-intensive and can fail for many microbes. Microscopic observation is also used to estimate abundance of microbes, and to a limited extent, assigns microbes to taxa [12]. Recently, 16S ribosomal RNA gene (rRNA) sequence profiles are used to elucidate microbial diversity, often to the phylotype level. Using 16S rRNA sequencing, microbes from mouth [13], [14], esophagus [15], stomach [16], small intestine [17], colon [18], [19] and vagina [20] have been studied. These studies explored microbial diversity within the human body and revealed a vast population of uncultivated and uncharacterized microbes, which had been elusive for cultivation-based methods. Through these high-throughput 16S rRNA sequencing and other metagenomic sequencing efforts, microbiota perturbations were found to be associated with periodontal disease [21] and obesity [22], [23], [24].

In the stomach, gastric acidity kills many ingested microbes. It was generally considered that the stomach is not inhabitable by any microbe until the discovery of *Helicobacter pylori* and its association with gastritis and peptic ulcer [25]. Other than a few other *Helicobacter* species [26], [27], [28], it was not expected that the stomach would contain many other live microbes. Reduced acidity due to progressive atrophic gastritis may increase microbial diversity [29]. A study by Monstein *et al.* using temporal temperature gradient gel electrophoresis (TTGE) and a small-scale 16S rRNA sequencing suggested other microbes such as *Enterococcus*, *Pseudomonas*, *Streptococcus*, *Staphylococcus* and *Stomatococcus* were also present in the stomach [30]. A more recent large-scale 16S rRNA sequencing effort identified 128 phylotypes from 8 phyla in 23 North American patients [16]. Interestingly, the presence of *H. pylori* in the stomach did not affect the overall composition of the microbiota at the phylum level.

In this study, we went further to investigate potential associations between stomach microbiota changes and Non-*H. pylori* and non-NSAID (NHNN) gastritis. We hypothesized that when *H. pylori* is not present, other bacterial groups/species may contribute to or be associated with gastritis development. On the microbiota level, we also would like to address a key issue in the field: at what taxon depth(s) does the microbiota appear relatively stable such that perturbations at these levels may be relevant to human health? We used 16S rRNA gene cloning and sequencing to profile the stomach microbiota from normal and NHNN gastritis patients, and taxon-specific real-time quantitative PCR (qPCR) assays to quantify the relative abundance of the Firmicutes phylum and the *Streptococcus* genus.

## Results

### Taxon tree analysis

We analyzed both body and antrum biopsies from 5 normal individuals and 5 NHNN antral gastritis individuals (all females, age-matched). All patients were *H. pylori* negative by both rapid urease test and 16S rRNA sequencing. None of the patients had taken NSAID within 6 months prior to undergoing endoscopy. At least 60 clones from each biopsy (body or antrum, thus at least 120 clones from each individual) were sequenced using broad-range 16S rRNA PCR products. A total of 1223 non-*H. pylori* microbial sequences were obtained. These microbes belong to 8 phyla (133 phylotypes), of which 5 phyla (Firmicutes, Bacteroidetes, Actinobacteia, Fusobacteria and Proteobacteria) are present in a vast majority (1211 out of 1223, or 99.0%). Nine phylotypes with sequence similarity less than 97% to sequences present in the public databases were identified. Six of these 9 phylotypes were represented by single clones (Supplementary Table S1).

**Figure 1:**
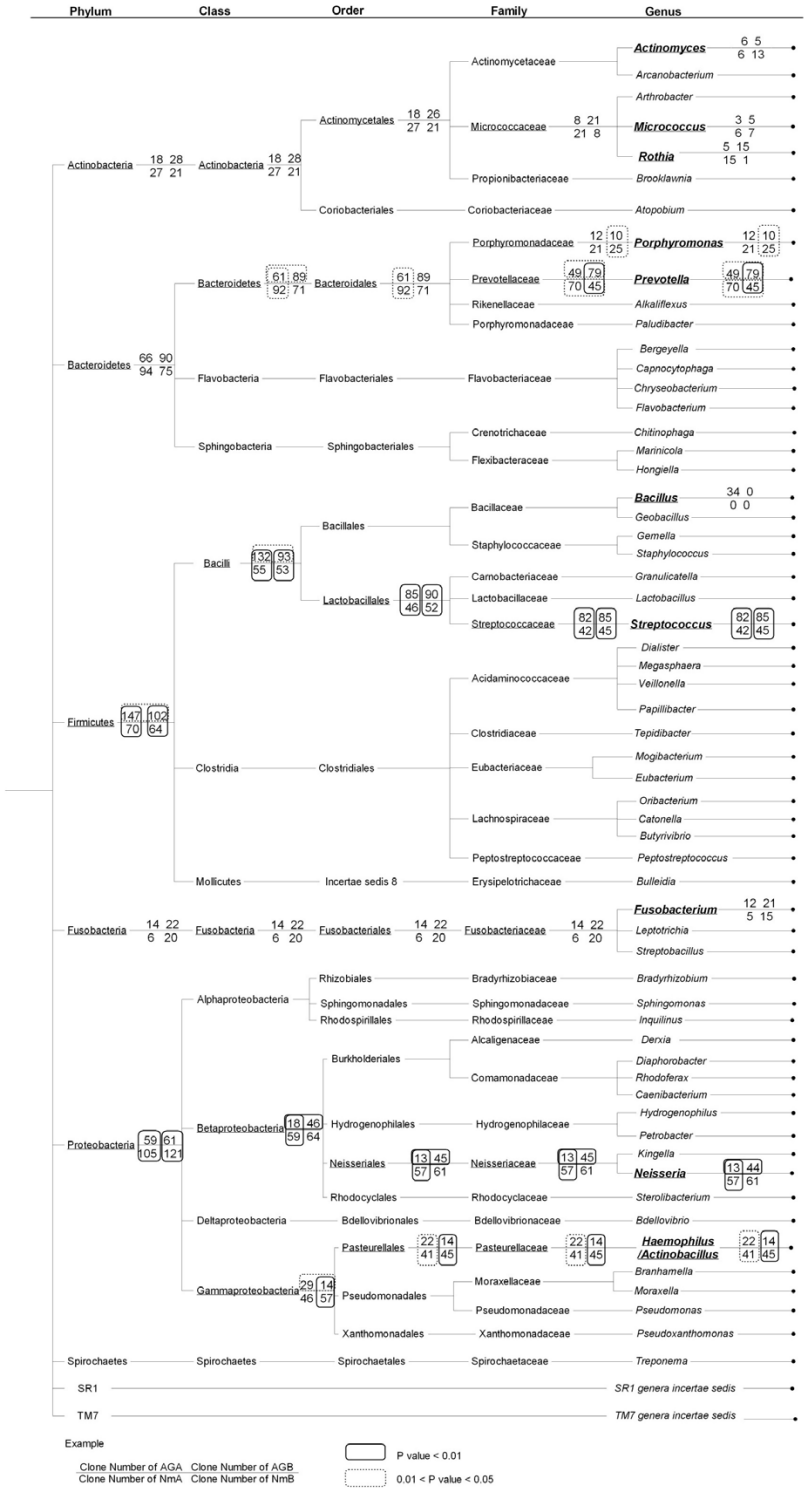
The taxon tree for the stomach microbiota. The tree was created based on the RDPII classifier based on a naive Bayesian rRNA classifier. The clone numbers for the top 5 phyla, top 6 classes, top 6 orders, top 7 families and top 10 genera were shown. Each taxon with at least 10 clones in all four sample groups (AGA, AGB, NmA, NmB) was analyzed by Pearson's chi-square test by comparing the clones numbers between AGA and AGB, NmA and NmB, AGA and NmA, and AGB and NmB. The clone numbers for all phylotypes were shown in Supplementary Figure S1.

To investigate the overall representation of different taxon levels in the stomach biota, we constructed a taxon tree (Figure 1 and supplementary Figure S1). Interestingly, each phylum was dominated by only one or two lower taxon levels (class, order, family or genus). As a matter of fact, each phylum was dominated by only 1–2 genera. For example, the most abundant phylum Firmicutes was represented by 383 clones, of those 333 clones are from the class Bacilli. Subsequently, 273 clones were from the order Lactobacillales. Two hundred and fifty four clones were from the family Streptococcaceae. And all 254 clones were from the genus *Streptococcus*. The five most common genera including *Streptococcus* (254 clones), *Prevotella* (243), *Neisseria* (175), *Haemophilus* (122), *Porphyromonas* (68), constituted 70.5% of all microbial clones.

### Species richness and diversity

When the entire dataset (1223 clones) was used, Good's coverage was 96%, indicating that four additional phylotypes would be expected for every 100 additional clones to be sequenced. This level of coverage indicated that majority of bacterial sequences were present in the sequenced clones. Diversity estimation by EstimateS version 8.0 indicated that about 200 phylotypes may be present in the human stomach biopsy samples (Supplementary Figure S2). We further estimated the species richness in four different biopsy samples (NmA (Normal Antrum), NmB (Normal Body), AGA (Antral Gastritis Antrum), AGB (Antral Gastritis Body); Supplementary Table S2). Species richness was not different between the antral gastritis biopsies and normal biopsies (p>0.1, unpaired t-test).

### Bacterial microbiota comparison between two different anatomical locations (antrum and body) in normal patients

In the normal patients, no significant difference was observed between two anatomical locations (antrum and body) for any of the taxon groups, except for the family Prevotellaceae and the genus *Prevotella* where the p values (Pearson's chi-square test) were between 0.01 to 0.05 (Figure 1).

### Firmicutes phylum and *Streptococcus* genus were enriched in the stomach of antral gastritis patients

**Figure 2:**
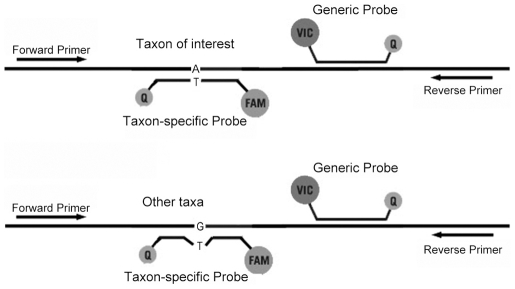
Taxon-specific qPCR for quantifying abundance of a specific taxon within the stomach microbiota. Two fluorescence probes (FAM and VIC) are located within the same PCR amplicon. The generic probe (VIC) targeting all bacteria sequences was used to quantify the total bacteria quantity. The taxon-specific probe (FAM) targeting to a specific taxon was used to quantify the quantity of the specific taxon. The taxon-specific probe matches perfectly to most species with the specific taxon, but has one or more mismatches to other species outside of the specific taxon. The ddCt value between VIC and FAM was used to quantify the abundance of the specific taxon.

Based on the 1223 16S rRNA sequences, the Firmicutes phylum was the most abundant phylum with 383 clones. The Proteobacteria phylum was a close second with 345 clones. Interestingly, the Firmicutes phylum was more abundant in antral gastritis biopsies than in normal biopsies (41% vs. 22%, Table 1), while the Proteobacteria phylum was more abundant in normal biopsies (37% vs. 20%). Since 16S rRNA sequencing is cost prohibitive for a larger sample size, we developed a taxon-specific real-time quantitative PCR (qPCR) approach to quantify the abundance of Firmicutes and *Streptococcus* (Figure 2). The taxon-specific qPCR data for Firmicutes were highly correlated with the 16S rRNA sequencing data for the aforementioned 20 biopsy samples (2 biopsies for each of the 5 normal and 5 antral gastritis patients) (Supplementary Figure S3).

**Table 1:** Clone numbers and percentages for each bacteria phylum in the gastric biopsies from 5 pairs (antrum and body) of normal and 5 pairs of antral gastritis patients.

Sample	Firmicutes	Proteobacteria	Bacteroidetes	Actinobacteria	Fusobacteria	Other	Total Clone #
**normal**							
B07A	13, 21%*	10, 16%	28, 45%	8, 13%	2, 3%	1, 2%	62
B07B	9, 15%	17, 27%	20, 32%	15, 24%	0, 0%	1, 2%	62
B14A	11, 18%	9, 15%	29, 48%	10, 17%	1, 2%	0, 0%	60
B14B	16, 26%	15, 24%	23, 37%	2, 3%	6, 10%	0, 0%	62
B19A	12, 19%	16, 26%	20, 32%	9,15%	1, 2%	4, 6%	62
B19B	14, 23%	20, 33%	17, 28%	3, 5%	4, 7%	2, 3%	60
B33A	16, 26%	43, 70%	1, 2%	0, 0%	1,2%	0, 0%	61
B33B	14, 23%	34, 57%	4, 7%	1, 2%	7, 12%	0, 0%	60
B34A	18, 29%	27, 44%	16, 26%	0, 0%	1, 2%	0, 0%	62
B34B	11, 18%	34, 56%	12, 20%	0, 0%	3, 5%	1, 2%	61
**Average**	22%	37%	28%	8%	4%	1%	100%
**antral gastritis**							
B10A	31, 52%	6, 10%	21, 35%	2, 3%	0, 0%	0, 0%	60
B10B	33, 54%	2, 3%	23, 38%	2, 3%	0, 0%	1, 2%	61
B16A	33, 54%	7, 11%	7, 11%	7, 11%	7, 11%	0, 0%	61
B16B	18, 29%	8, 13%	23, 37%	7, 11%	5, 8%	1, 2%	62
B22A	16, 26%	16, 26%	25, 40%	0, 0%	5, 8%	0, 0%	62
B22B	16, 25%	16, 25%	23, 37%	1, 2%	7, 11%	0, 0%	63
B28A	26, 43%	17, 28%	11, 18%	5, 8%	2, 3%	0, 0%	61
B28B	15, 25%	17, 29%	15, 25%	9, 15%	3, 5%	0, 0%	59
B35A	41, 68%	13, 22%	2, 3%	4, 7%	0, 0%	0, 0%	60
B35B	20, 32%	18, 29%	6, 10%	9, 15%	7, 11%	2, 3%	62
**Average**	41%	20%	25%	8%	6%	1%	100%

* Clone number, percentage.

**Figure 3:**
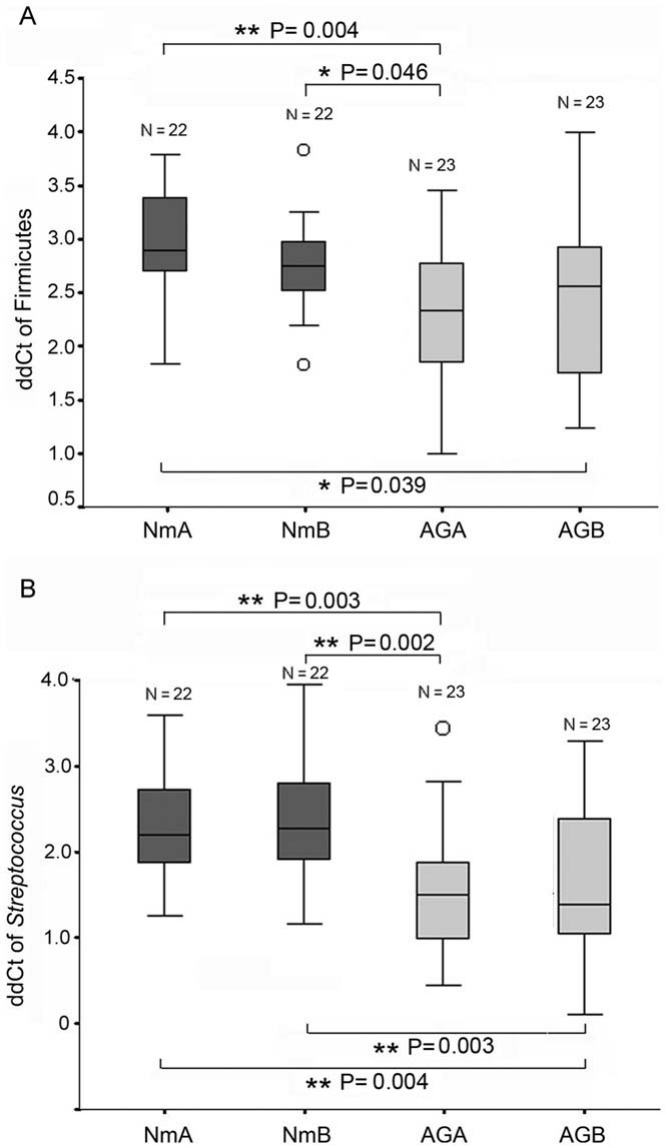
Abundance of the Firmicutes phylum and the *Streptoccous* genus in different biopsies. Delta delta Ct (ddCt) values (see text for details) were used to represent the abundance of the two taxa. A higher ddCt value represents a lower abundance of the taxon (abundance = 2^−ddCt^).

Seventeen additional pairs of antrum and body biopsy samples from normal patients and 18 additional pairs of antrum and body biopsy samples from antral gastritis patients were analyzed by Firmicutes-specific qPCR. Totally, 90 biopsies (46 samples from 23 antral gastritis patients and 44 samples from 22 normal patients) were analyzed (Table 2). The mean age of the antral gastritis patients was 67.6±11.4 (median: 69, range: 46 to 86), while the mean age of the controls was 58.3±14.7 (median: 52, range: 40 to 87). The mean age of the normal group was younger than that of the antral gastritis group. But statistical analysis showed that there was no correlation between age and Firmicutes or *Streptococcus* abundance (p>0.1) (Supplementary Figure S4). These samples were divided into 4 groups, antral gastritis antrum (AGA), antral gastritis body (AGB), normal antrum (NmA), and normal body (NmB). The abundance of Firmicutes was significantly higher in AGA than in NmA or NmB (One-way ANOVA (Analysis of Variance) test, p = 0.004 and p = 0.046, respectively) and in AGB than in NmA (one way ANOVA, p = 0.039) (Figure 3A). No significant difference was observed between AGA and AGB (one way ANOVA, p = 0.855), or NmA and NmB (p = 0.832).

**Table 2:** Patient demographics.

NO.	Sex	Age	RUT	Disease Status	Gastric atrophy
P007	F	49	-ve*	NA (endoscopically normal)	No
P012	M	47	-ve	NA (endoscopically normal)	No
P014	F	78	-ve	NA (endoscopically normal)	No
P019	F	57	-ve	NA (endoscopically normal)	No
P033	F	74	-ve	NA (endoscopically normal)	No
P034	F	68	-ve	NA (endoscopically normal)	No
P039	M	56	-ve	NA (endoscopically normal)	No
P050	M	51	-ve	NA (endoscopically normal)	No
P059	F	67	-ve	NA (endoscopically normal)	No
P065	F	46	-ve	NA (endoscopically normal)	No
P073	F	40	-ve	NA (endoscopically normal)	No
P081	F	42	-ve	NA (endoscopically normal)	No
P086	F	50	-ve	NA (endoscopically normal)	No
P087	F	47	-ve	NA (endoscopically normal)	No
P091	F	52	-ve	NA (endoscopically normal)	No
P105	M	82	-ve	NA (endoscopically normal)	No
P106	F	46	-ve	NA (endoscopically normal)	No
P302	F	81	-ve	NA (endoscopically normal)	No
P305	F	42	-ve	NA (endoscopically normal)	No
P338	F	68	-ve	NA (endoscopically normal)	No
P383	F	87	-ve	NA (endoscopically normal)	No
P453	F	52	-ve	NA (endoscopically normal)	No
P010	F	72	-ve	HP Neg mild gastritis (endoscopically antral gastritis)	No
P016	F	86	-ve	HP Neg mild chronic antral gastritis	No
P022	F	55	-ve	endoscopically antral gastritis	No
P028	F	65	-ve	HP Neg mild chronic antral gastritis	No
P035	F	78	-ve	HP Neg mild gastritis (endoscopically antral gastritis)	No
P040	M	78	-ve	HP Neg mild gastritis (endoscopically antral gastritis)	No
P045	M	75	-ve	HP Neg mild gastritis (endoscopically antral gastritis)	Yes
P062	F	63	-ve	HP Neg mild chronic antral gastritis	No
P067	F	72	-ve	HP Neg mild gastritis (endoscopically antral gastritis)	Yes
P074	F	68	-ve	HP Neg mild gastritis (endoscopically antral gastritis)	No
P076	F	68	-ve	HP Neg mild gastritis (endoscopically antral gastritis)	No
P078	M	46	-ve	endoscopically antral gastritis	No
P089	F	48	-ve	HP Neg mild chronic antral gastritis	No
P100	M	69	-ve	HP Neg mild gastritis (endoscopically antral gastritis)	No
P103	F	72	-ve	endoscopically antral gastritis	No
P109	F	71	-ve	HP Neg mild chronic antral gastritis	No
P110	M	60	-ve	endoscopically antral gastritis	No
P111	F	84	-ve	HP Neg mild gastritis (endoscopically antral gastritis)	No
P114	M	60	-ve	endoscopically antral gastritis	No
P301	F	74	-ve	HP Neg mild chronic antral gastritis	No
P306	F	54	-ve	HP Neg mild chronic antral gastritis	No
P451	F	52	-ve	HP Neg mild chronic antral gastritis	No
P452	M	85	-ve	HP Neg mild chronic antral gastritis	No

* RUT test negative.

For the same biopsy samples, the genus *Streptococcus* was also analyzed by *Streptococcus*-specific qPCR. *Streptococcus* abundance was 72% and 76% higher in AGA vs. NmA or NmB, respectively, and 66% and 70% higher in AGB vs. NmA or NmB, respectively (Figure 3B). The p values for ANOVA test are shown in Figure 3B. Similar to the Firmicutes assay, no significant difference was observed between AGA and AGB (one way ANOVA, p = 0.999), or NmA and NmB (p = 0.999).

### *Streptococcus* cultivation and biopsy washing

The mere detection of 16S rRNA gene sequences does not imply that live bacteria are present or the bacteria are indeed resident instead of passersby in the stomach. We thus carried out two additional experiments.

Firstly, we attempted bacteria cultivation from the biopsies. A culture condition suitable for *Streptococcus* was used since they appeared to be over-represented in antral gastritis patients. Sixteen pairs (antrum and body) of biopsies were used for culture on the blood agar plates. Colonies were sequenced for the 16S rRNA gene for identification. Eleven phylotypes of *Streptococcus* were isolated (Supplementary Table S3). These 11 phylotypes constituted 93.3% (or 237 out of the 254 clones) of all the clones identified in the broad-range 16S rRNA sequencing, indicating that majority of the *Streptococcus* phylotypes are alive in the gastric biopsies.

Secondly, 14 biopsy samples from both antral gastritis and normal patients were washed in phosphate buffered saline (PBS) with three increasingly harsh conditions. If harsh washing conditions do not remove the bacteria from the biopsy, it is suggestive that these bacteria attach closely to the stomach mucosa. Over 90% of the total bacteria remained attached to the biopsies after three consecutive washings with increasingly harsh conditions (Supplementary Figure S5A). The last washing step was done on high power on a desktop vortex machine. Similarly, majority of the *Streptococcus* bacteria remained attached to the biopsies after the 3 washing steps (Supplementary Figure S5B).

## Discussion

In this study, we have profiled the bacterial microbiota in the paired gastric biopsies (antrum and body) from normal and antral gastritis patients. All patients are *H. pylori* negative and without NSAID use. Through broad-range 16S rRNA gene sequencing, we identified 1,223 non-*H pylori* bacteria clones, similar to a previous study (Bik study, 1,056 non-*H pylori* bacteria clones) [16]. Although the two studies analyzed two geographically (Hong Kong vs. California) and ethnically (Chinese vs. Caucasian, Hispanics and African American) divergent populations, the overall microbiota complexities are surprisingly similar (Table 3). Both studies identified approximately 130 (133 and 127 for this and the Bik study, respectively) phylotypes from seven to eight phyla. Majority of the clones (77.4% of this study and 79.8% of Bik study) were shared. The two most abundant genera (*Streptococcus* and *Prevotella*) were also identical. These two genera represented 40.6% and 41.5% of all clones in this study and the Bik study. Both studies indicated that approximately 200 different phylotypes may be present in the stomach mucosa. Such dramatic similarity between the two studies highlights the selective pressure for the microbiota under the harsh stomach environment. Additionally, we found little difference in bacterial microbiota between the two anatomical sites (antrum and body) in normal patients, despite of the clinical relevance for biopsy sampling at different anatomical sites [31].

**Table 3:** Comparison between this study and the Bik study.

	This study	Bik study
**Patients/Samples**	10/20	23/23
**Clone No. (non ** ***H. pylori*** **)**	1223	1056
**Phyla**	8	7
**Total Phylotypes**	133	127
**New Phylotypes**	9	13
**Phylotypes in both studies (clone number, percentage)**	59 (946, 77.4%)	59 (843, 79.8%)
**Study Specific Phylotypes (clone number, percentage)**	74 (277, 22.6%)	68 (213, 20.2%)
**Top 5 genera (clone number)**	***Streptococcus*** ** (254)**	***Streptococcus*** ** (299)**
	***Prevotella*** ** (243)**	***Prevotella*** ** (139)**
	*Neisseria* (175)	*Rothia* (95)
	*Haemophilus* (122)	*Fusobacterium* (45)
	*Porphyromonas* (68)	*Veillonella* (41)

Given that rigorous washing steps were not able to separate the microbiota from the biopsies, we hypothesize that majority of the identified bacteria are associated tightly with the stomach mucosa. Additionally, we were able to cultivate majority of the *Streptococcus* phylotypes identified through broad-range 16S rRNA sequencing, suggesting that these bacteria may indeed be true residents in the stomach mucosa.

To further appreciate the overall complexity of stomach microbiota, we constructed a taxon tree based on the identified clones to look into each taxon level including phylum, class, order, family and genus. Interestingly, we found that for each phylum, one or two genera were predominantly present. The five most common genera including *Streptococcus* (phylum Firmicutes), *Prevotella* and *Porphyromonas* (Bacteroidetes), as well as *Neisseria* and *Haemophilus* (Proteobacteria), constituted 70.5% of all microbial clones.

Interestingly, the 16S rRNA profiling revealed a significant over-representation of the Firmicutes phylum (primarily due to the over-representation of the *Streptococcus* genus within the phylum) and an under-representation of the Proteobacteria phylum in the biopsies from antral gastritis patients. We developed a taxon-specific qPCR approach to analyze the abundance of the Firmicutes and *streptococcus* taxa for 90 biopsies (46 samples from 23 antral gastritis patients and 44 samples from 22 normal patients) and confirmed the over-representation of these two taxa in antral gastritis stomach by 42% and 71%, respectively. Most of the *Streptococcus* phylotypes identified by sequencing were alpha-hemolytic bacteria that are potential pathogens (e.g. *Streptococcus pneumoniae*, *Streptococcus mitis* and *Streptococcus salivarius*). Certain *Streptococcus* species are resistant to low pH conditions and may survive in the stomach [32]. Our cultivation data and washing experiment also suggested that these were indeed living, resident biota in the stomach.

Whether the increase in *Streptococcus* abundance is causative for antral gastritis or a result of local environmental change due to antral gastritis remained to be answered. One potential approach is using germ-free mouse model [33]. Another intriguing question is that whether certain microbiota compositions protect, or alternatively, sensitize the stomach mucosa from invading pathogens such as *H. pylori*. Lastly, new high throughput sequencing technologies are likely to provide more comprehensive data on microbiota from different anatomical locations along the human digestive tract and at different time points [34].

## Materials and Methods

### Gastric biopsy samples

This study was approved by the Chinese University of Hong Kong clinical research ethics committee. All patients gave written informed consent for obtaining the study specimens. Two gastric mucosal biopsies (antrum and body of the stomach) were collected from each patient during routine endoscopy at the Prince of Wales Hospital, Hong Kong. To avoid contamination, a new sterilized endoscopy forceps was used when taking a second biopsy from the same patient. The biopsies were snap-frozen on dry ice and stored at −80°C. Patients taking antibiotics or NSAIDs (defined as any use of NSAID for at least one week in the past 3 months prior to endoscopy) or tested positive for *H. pylori* by rapid urease test (RUT) or histological test were excluded. Patient demography is shown in Table 2.

### Construction of 16S rRNA clone libraries and sequencing

Total genomic DNA was isolated from the biopsies by using the DNA mini kit (Qiagen, Valencia, CA, USA) with glass beat beater method as previously described [16]. Two negative controls with only sterile water were also extracted using the same protocol. The extracted DNA concentrations were measured by NanoDrop 1000 Spectrophotometer (Thermo Scientific, Minneapolis, MN, USA). Two universal bacterial 16S rRNA primers, B8F20 [35] (5′-AGAGTTTGATCCTGGCTCAG-3′) and B806R20 [36] (5′- GGACTACCAGGGTATCTAAT-3′) were used to amplify the region corresponding to position 8 to 806 of the *Escherichia coli* 16S rRNA gene. The 25 µL PCR mixtures included 1× PCR buffer including 1.5 mM MgCl_2_ (Qiagen), 20 mM tetramethylammonium chloride, 0.1 mM of each dNTP, 0.4 µM of each primer, 1 unit of HotStar Taq DNA polymerase (Qiagen), and 2 µL extracted DNA. A thirty-cycle PCR was performed to amplify the 799 bp fragment. The PCR products were checked by agarose gel electrophoresis. For each product, a single band could be observed under UV light, while no band was seen for the negative controls. The 16S rRNA products were purified with Sephadex G-50 column (Sigma-Aldrich, St. Louis, MO, USA), ligated with T vectors and transformed into *E. coli* JM109 cells by using the pGEM-T easy vector system (Promega, Madison, WI, USA). We selected 5 patients (10 biopsy samples) with antral gastritis and 5 normal controls (10 biopsy samples) to construct 20 16S rRNA gene libraries. For each gastric biopsy library, at least 60 colonies were selected for sequencing. The PCR products were sequenced by using BigDye terminator v3.1 cycle sequencing kit (Applied Biosystems, Foster City, CA, USA). The sequencing reactions using B8F20 as the sequencing primer were carried out on a ABI 3730xl sequencer (Applied Biosystems).

### Phylogenetic analysis and microbial diversity estimation

The chimeric test using the Bellerophon server (http://foo.maths.uq.edu.au/~huber/bellerophon.pl) [37] was used to test potential chimeric sequences. One clone was found to be chimeric and subsequently excluded. Then, the 1223 nonchimeric sequences were analyzed by RDP II (Ribosomal Database Project II) classifier (http://rdp.cme.msu.edu/classifier/classifier.jsp) based on a naive Bayesian rRNA classifier [38]. Basic local alignment search tool (BLAST) provided by Green Genes (http://greengenes.lbl.gov/cgi-bin/nph-blast_interface.cgi) was performed to find the most similar sequences in the database. We used 97% sequence identity as the cutoff for defining phylotypes [39]. Sequences with identity <97% to the existing sequences in the database were considered novel. The taxon tree was constructed by using the classification result of the RPD II classifier. Chao1 estimator of the EstimateS 8 program (http://viceroy.eeb.uconn.edu/estimates) was used to estimate the microbial diversity. Good's method was used to calculate sequencing coverage [40].

### Real-time quantitative PCR (qPCR)

Q-PCR primers and probes were designed based on the sequences obtained from the cloned libraries. We first aligned all cloned sequences by ClustalW (http://www.ebi.ac.uk/Tools/clustalw2/index.html) with the default parameters. The qPCR primers were B8F20 and B801R21 (5′- ACCAGGGTATCTAATCCTGTT-3′). The MGB probe sequences are: generic probe (VIC) 5′- CAGCAGCCGCGGTAA-3′, Firmicutes probe (FAM) 5′- AAGATTCCCTACTGCTGCCT-3′ and *Streptococcus* probe (FAM) 5′- TACACATGGAATTCCAC-3′. To measure the abundance of a specific taxon, two probes (one specific for the taxon of interest and the second generic for all bacteria) were used in the same PCR amplicon. (Figure 2). The 25 µL PCR mixture included 1× Buffer A, 3.5 mM MgCl_2_, 200 µM dNTP with dUTP instead of dTTP, 400 nM of each primer, 100 nM of each probe, 0.01 U/µL Uracil-N-Glycosylase, and 0.05 U/µL TaqGold (Applied Biosystems). For the Firmicutes-specific assay, the cycling condition was: 1) 50°C for 2 min; 2) 95°C for 10 min; 3) 40 cycles of 95°C for 20 sec, 58°C for 15 sec, 70°C for 80 sec. For the *Streptococcus*-specific assay, the cycling condition was: 1) 50°C for 2 min; 2) 95°C for 10 min; 3) 40 cycles of 95°C for 20 sec, 57°C for 1 min, 70°C for 1 min. The *Streptococcus* 16S rRNA fragment (DQ346438) cloned into the pGEM-T easy vector was used as the standard for qPCR (both for the Firmicutes and the *Streptococcus* assays) in the ABI 7500 real-time PCR system (Applied Biosystems). Due to some slight difference between the generic and taxon-specific probes, delta delta threshold cycle (ddCt) was used to indicate the abundance of the specific taxon in the entire bacteria population.
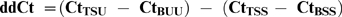
(Ct_TSU_: Ct of taxon specific probe from unknown samples, Ct_BUU_: Ct of bacterial universal probe from unknown samples, Ct_TSS_: Ct of taxon specific probe from the plasmid standard, Ct_BSS_: Ct of bacterial universal probe from the plasmid standard)

Theoretically, the abundance of a taxon is 2^−ddCt^.

### *Streptococcus* Cultivation

We obtained 32 additional biopsies from 16 patients for bacterial culture. The biopsies were put in phosphate buffered saline (PBS, pH = 7.2) and cut to smaller pieces by a scalpel. The samples were then spread on blood agar plates (CM331, Oxoid, Basingstoke, UK) with 5% horse blood. The plates were placed in a 5% CO_2_ incubator at 37°C for 24 hours. The colonies with hemolysis on the blood agar were picked for 16S rRNA sequencing.

### Biopsy washing

For biopsy washing test, 14 additional samples from both antral gastritis patients and normal people were collected. Each sample was placed in a 2.0 mL tube and washed 3 times (200 µL PBS for each washing) under increasingly harsh conditions. The first washing was done by gentle hand shaking. The supernatant was transferred out. New PBS was added to the biopsies for further washing. For the second and third washing, the tubes were vortexed by the tube mixer Trio TM-2F (All-lab scientific, AU) at grade 3 and grade 6 power level respectively, roughly corresponding to gentle and vigorous vortexing. Then duplex real-time PCR was performed to test the total bacteria and the *Streptococcus* quantities in the PBS supernatants from the three washing steps and the washed biopsies.

### Statistics analysis

Pearson's chi-square test was used to compare the clone numbers of different taxa between different sample groups (NmA: normal antrum, NmB: normal body, AGA: antral gastritis antrum, AGB: antral gastritis body) from the 16S rRNA sequencing result, when the clone number for each sample group was at least 10. ANOVA test was used to compare the bacterial abundance data from qPCR assays. All analyses were performed using SPSS for windows, version 11.5 (SPSS Inc., Chicago, IL, USA). P<0.05 was considered statistically significant. For comparing the species richness between normal and antral gastritis patients, the actual phylotype number for each patient was counted first. Unpaired t-test was then used to compare the two patient groups.

## Supporting Information

Figure S1 Detailed taxon tree
(2.00 MB TIF)

Figure S2 Species richness estimate
(0.97 MB TIF)

Figure S3 Correlation between qPCR and 16S rRNA cloning and sequencing
(0.98 MB TIF)

Figure S4 Lack of correlation between patient age and Firmicutes or Streptococcus abundance
(2.01 MB TIF)

Figure S5 Harsh washing does not remove the bacteria from the biopsies
(1.90 MB TIF)

Table S1 Novel phyltoypes
(0.03 MB XLS)

Table S2 Species richness estimate in different biopsy samples
(0.02 MB XLS)

Table S3 (0.02 MB XLS)
