# Supplementary figures and images for: Bacterial Microbiota Profiling in Gastritis without Helicobacter pylori Infection or Non-Steroidal Anti-Inflammatory Drug Use
Source: PLoS One. 2009 Nov 24;4(11):e7985. doi: 10.1371/journal.pone.0007985 (PMC2776972; doi:10.1371/journal.pone.0007985)

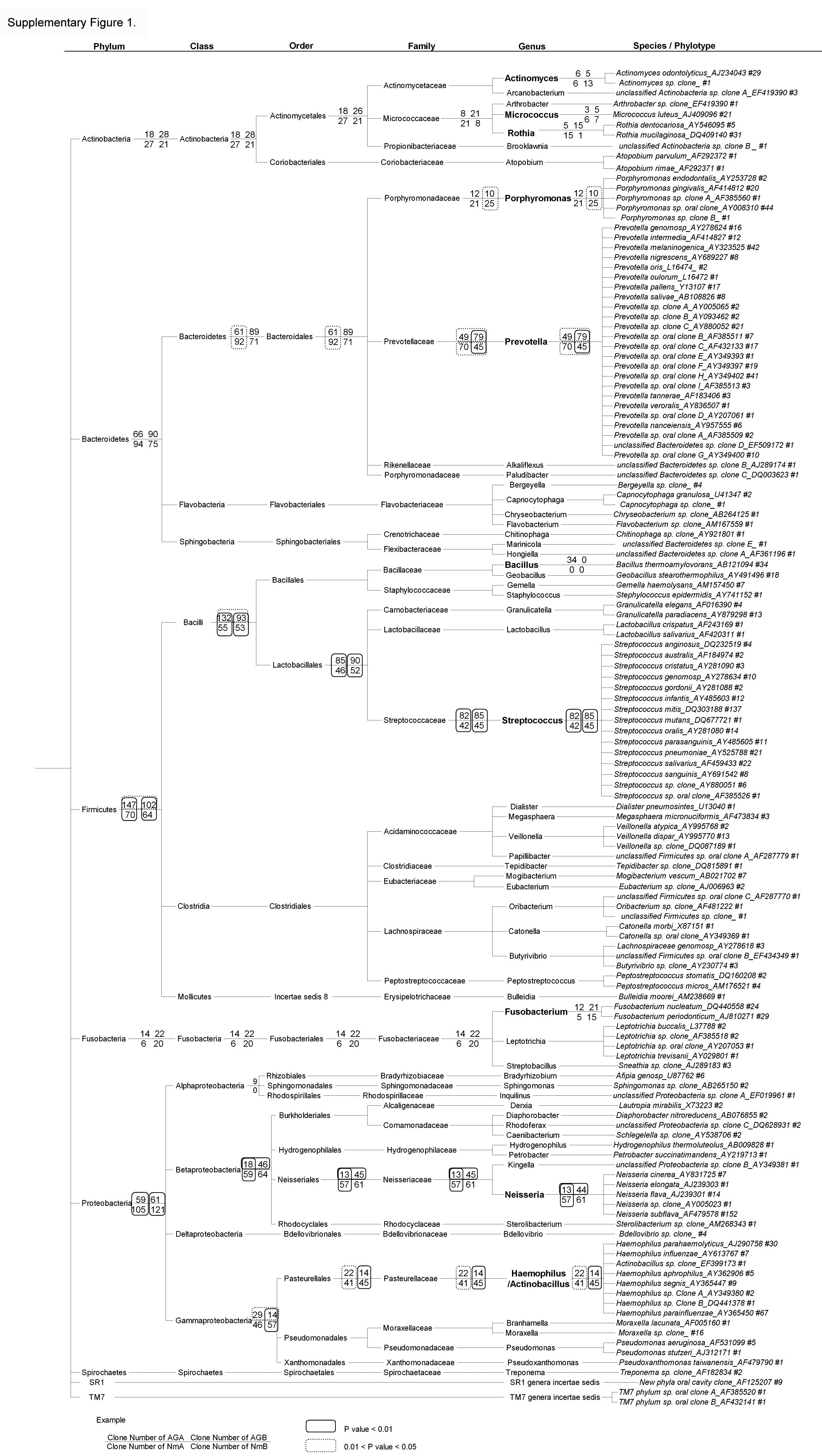

Supplement: Figure S1 — Detailed taxon tree (2.00 MB TIF) [file pone.0007985.s001.tif]

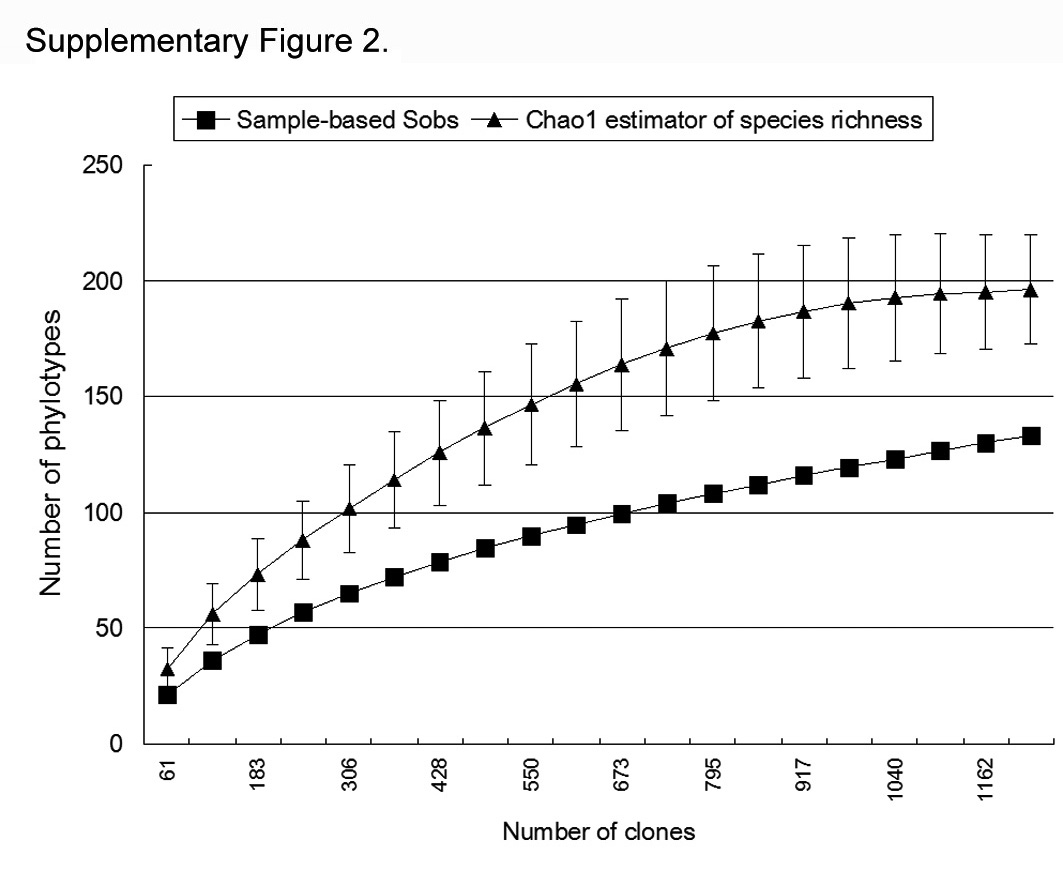

Supplement: Figure S2 — Species richness estimate (0.97 MB TIF) [file pone.0007985.s002.tif]

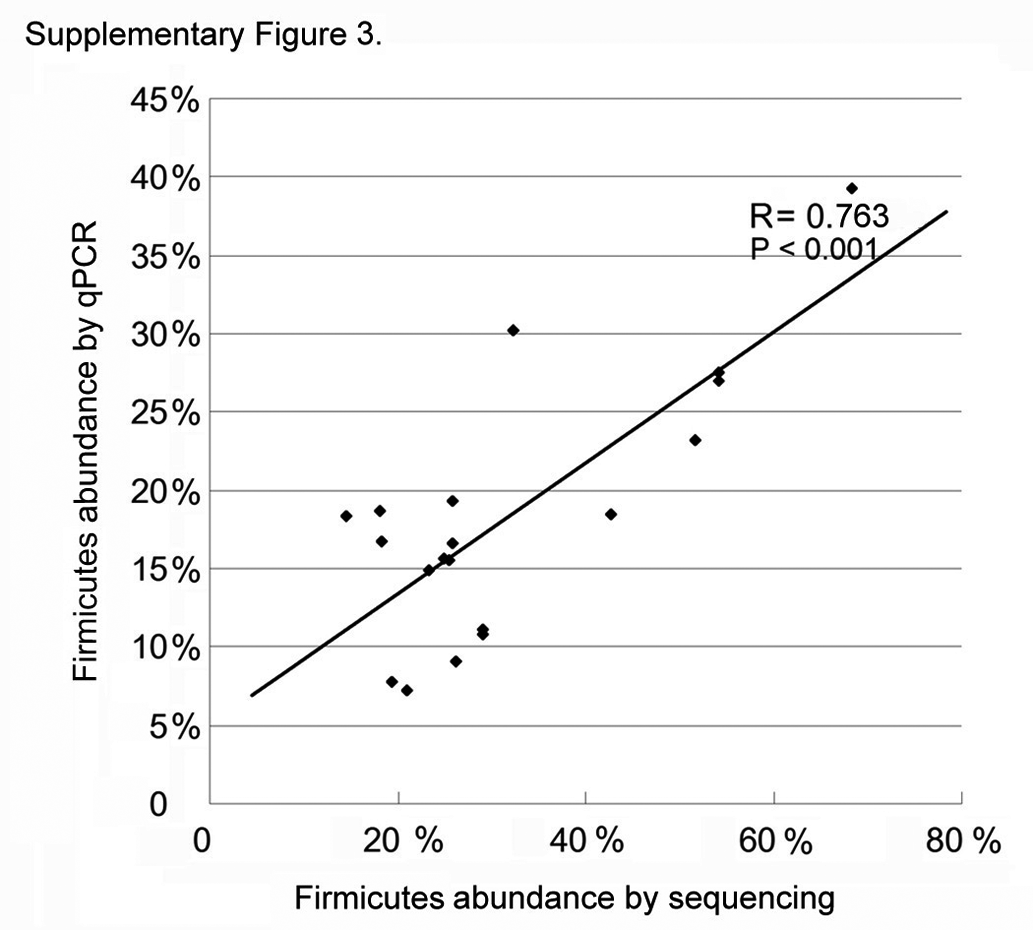

Supplement: Figure S3 — Correlation between qPCR and 16S rRNA cloning and sequencing (0.98 MB TIF) [file pone.0007985.s003.tif]

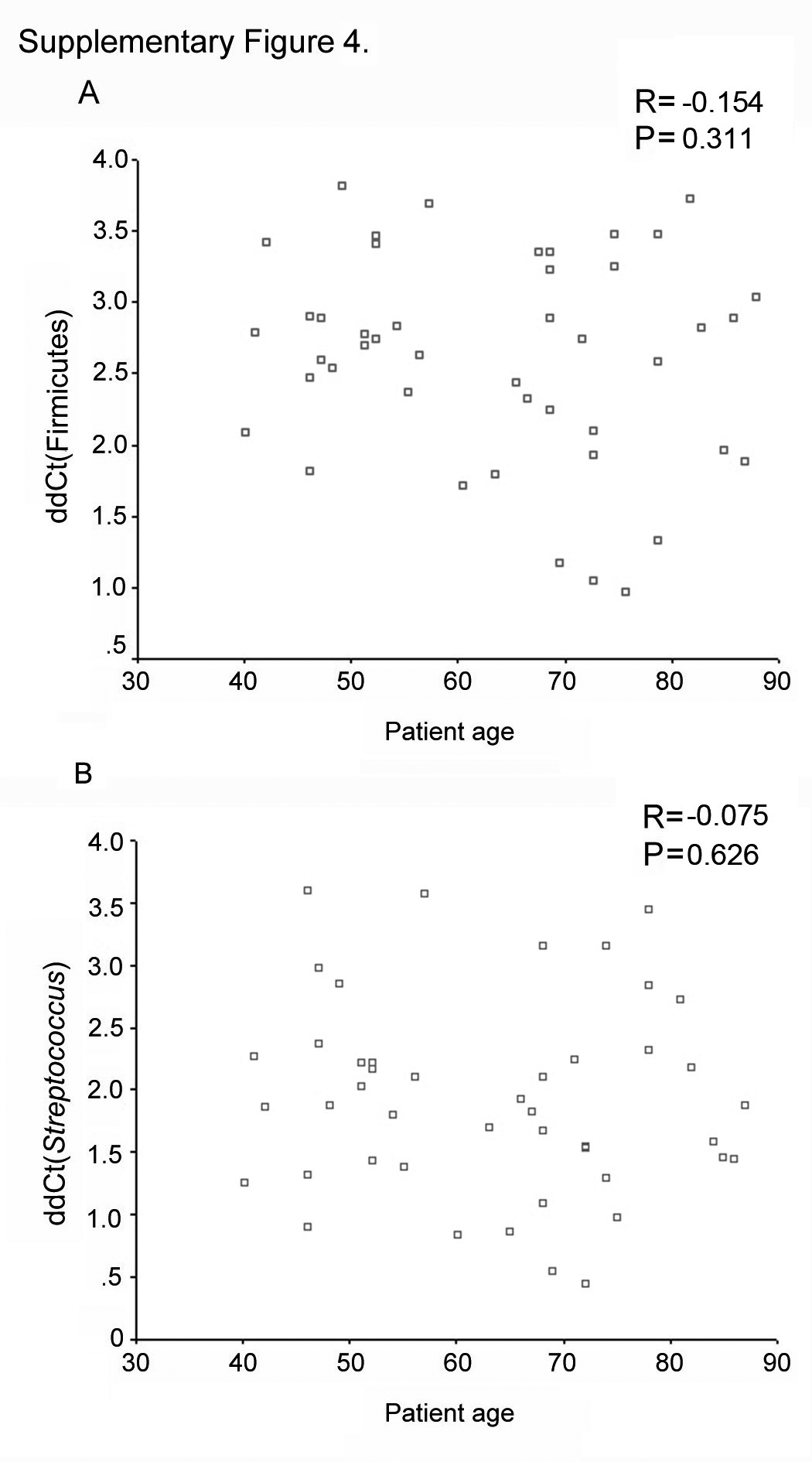

Supplement: Figure S4 — Lack of correlation between patient age and Firmicutes or Streptococcus abundance (2.01 MB TIF) [file pone.0007985.s004.tif]

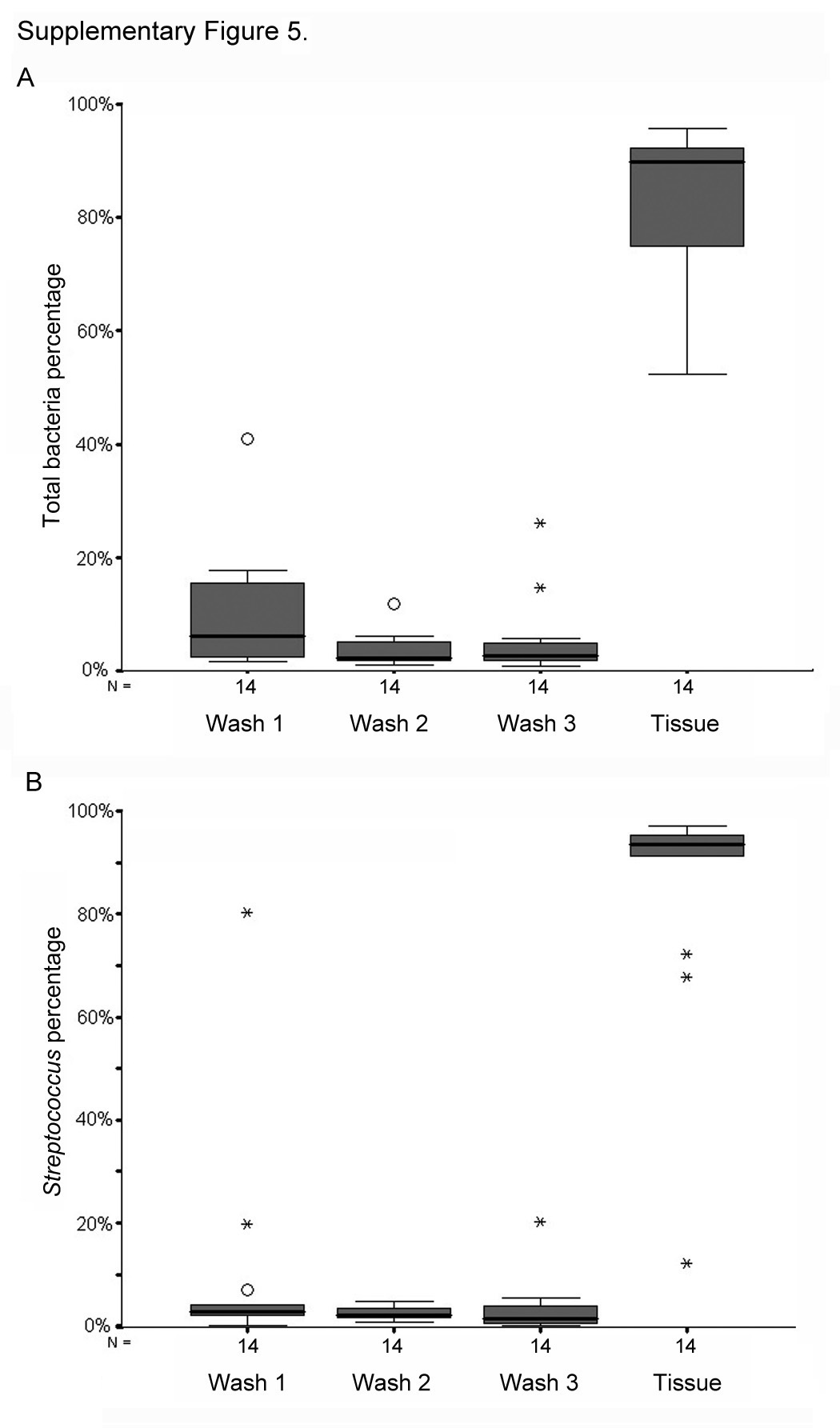

Supplement: Figure S5 — Harsh washing does not remove the bacteria from the biopsies (1.90 MB TIF) [file pone.0007985.s005.tif]
